# Association between coffee consumption and metabolic syndrome: A cross‐sectional and Mendelian randomization study

**DOI:** 10.1111/1753-0407.70004

**Published:** 2024-10-10

**Authors:** Tommy Hon Ting Wong, Shan Luo, Shiu Lun Au Yeung, Jimmy Chun Yu Louie

**Affiliations:** ^1^ School of Biological Sciences, Faculty of Science The University of Hong Kong Pokfulam Hong Kong SAR; ^2^ School of Public Health, Li Ka Shing Faculty of Medicine The University of Hong Kong Pokfulam Hong Kong SAR; ^3^ Department of Nursing and Allied Health, School of Health Sciences Swinburne University of Technology Hawthorn Victoria Australia

**Keywords:** artificial sweetener, coffee, Mendelian randomization, metabolic syndrome, milk, sugar

## Abstract

**Background:**

This study investigates the associations between coffee consumption and metabolic syndrome and its components, as well as the effect of milk, sugar, and artificial sweeteners on these associations.

**Methods:**

A cross‐sectional analysis was conducted with 351805 UK Biobank participants. Coffee consumption data were collected via food frequency questionnaires and 24‐h recall. Metabolic syndrome was identified through blood biochemistry and self‐reported medication use. Odds ratios were calculated using multivariable logistic regression, and results were verified with two‐sample Mendelian randomization.

**Results:**

Consuming up to two cups of coffee per day was inversely associated with metabolic syndrome (1 cup/day: odds ratio [OR]: 0.88, 95% confidence interval [CI]: 0.85–0.92; 2 cups/day: OR: 0.90, 95% CI: 0.86–0.93). Higher intakes showed near‐null associations. Mendelian randomization did not support a causal link between coffee intake and metabolic syndrome. Both self‐reported and genetically predicted high coffee consumption (four cups per day or more) were associated with central obesity. The inverse association between coffee consumption and metabolic syndrome was more profound among drinkers of ground coffee than those of instant coffee. Results were similar when stratified by the use of milk and sugar, yet the use of artificial sweetener with coffee was positively associated with metabolic syndrome and all component conditions.

**Conclusions:**

Coffee consumption may increase the risk of central obesity but is unlikely to impact the risk of metabolic syndrome. The potential health effects of artificial sweeteners in coffee need further investigation.

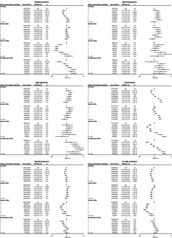

## Graphical abstract

Highlights

- Coffee consumption ≤2 cups/day inversely linked to metabolic syndrome (MetSyn).
- No causal relationship between coffee intake and MetSyn.
- High coffee consumption (4+ cups/day) associated with central obesity.
- Ground coffee showed stronger inverse association with MetSyn than instant coffee.
- Use of artificial sweetener with coffee linked to MetSyn and its components.

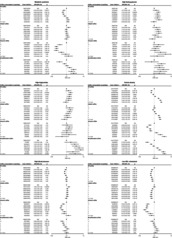

## 1 INTRODUCTION

Metabolic syndrome is characterized by a cluster of metabolic abnormalities, including central obesity, high blood pressure, dyslipidemia, and hyperglycemia.^1^ The presence of metabolic syndrome increases risk for type 2 diabetes, cardiovascular disease, and mortality.^2^, ^3^ With a global prevalence estimated between 20% and 30%, metabolic syndrome poses a major public health burden.^4^, ^5^, ^6^, ^7^ Identifying modifiable risk factors for metabolic syndrome is therefore critically important.

Some observational studies have reported an inverse association between coffee consumption and metabolic syndrome risk.^8^ Potential mechanisms include the beneficial effects of caffeine on energy balance^9^ and anti‐inflammatory effects of coffee polyphenols.^10^ However, evidence from randomized controlled trials does not support a causal effect of coffee on metabolic risk factors like glucose and lipids.^11^, ^12^ In addition, longitudinal studies suggest associations may be confounded by lifestyle and socioeconomic factors.

While coffee intake overall may not influence metabolic syndrome risk, differences by coffee type, as well as additives like milk, sugar, and sweeteners, have been scarcely studied. Detailed investigation of these exposures could provide novel insights.

Therefore, we aimed to examine associations of total coffee intake, coffee subtypes, and use of additives with metabolic syndrome and its components cross‐sectionally in a large cohort. A detailed understanding of these observational relationships can help inform future research into the metabolic effects of coffee. We also conducted a Mendelian randomization study, which used genetic variants allocated at conception and hence less vulnerable to confounding, to verify the observational association.^13^

## 2 METHODS

### 2.1 Study population

The present study utilized data from the UK Biobank, a large‐scale prospective cohort study conducted among middle‐to‐older adults across the United Kingdom. The design and the protocol of this project have been previously published.^14^, ^15^ Approximately 500 000 participants between 40 and 69 years were recruited from the general population at baseline during 2006–2010. Comprehensive assessments were undertaken at 22 centers throughout the UK to collect extensive data on participant demographics, socioeconomic status, medical history, lifestyle factors, health outcomes, and biological measures. Moreover, blood specimens were collected for biochemical assays and genotyping. All UK Biobank participants were genotyped using either the Affymetrix UK BiLEVE Axiom or Affymetrix UK Biobank Axiom array. Ethical approval was obtained from the North West Multi‐Centre Research Ethics Committee (reference number: 11/NW/0382), and written informed consent was secured from all participants before data collection. The present analyses were conducted under UK Biobank application number 44407.

### 2.2 Exposure assessment

Habitual coffee consumption was ascertained using both a food frequency questionnaire (FFQ) and repeated 24‐h dietary recalls collected at different time points. On the FFQ, participants reported their average daily coffee intake over the past year, measured in cups per day, with each cup defined as 200–250 mL. The FFQ also inquired about the intake of specific coffee subtypes including instant, ground, and decaffeinated, among others.

Additionally, subsets of participants completed up to five 24‐h dietary recalls. On each recall, participants reported their coffee consumption on the previous day, including intake in cups of various subtypes such as instant, filtered, and espresso. Further, the recalls captured additions to coffee such as milk, sugar, and artificial sweeteners.

### 2.3 Outcomes

The primary outcome was defined as metabolic syndrome, in accordance with the harmonized criteria set by the International Diabetes Federation.^1^ Specifically, participants meeting the threshold for central obesity based on population‐specific waist circumference cutoffs, along with any two of the following, were classified as having metabolic syndrome: elevated fasting glucose, blood pressure, triglycerides, and reduced high density lipoprotein (HDL)‐cholesterol. Detailed criteria for each condition were outlined in Table S1. Waist circumference was measured to the nearest centimeter during the physical assessment utilizing a SECA tape measure. Blood pressure was assessed twice via an automated Omron monitor, with the average of the two readings utilized in analyses. Fasting blood biomarkers were assayed using a Beckman Coulter AU5800 analyzer. Self‐reported medication use for treating dysglycemia, dyslipidemia, and hypertension was ascertained through touchscreen questionnaires. The corresponding medication codes were shown in Table S2.

### 2.4 Case and control ascertainment of outcomes

For each outcome, cases were defined as meeting the ascertainment criteria, while controls were defined as not meeting the ascertainment criteria without missing data. Handling of missing data was addressed in the next section. For metabolic syndrome, individuals meeting the criteria for cases in three or more of the component conditions were considered cases, while those meeting the criteria for controls in three or more of the component conditions were labeled as controls. Participants not meeting the criteria for either case or control were excluded from the analysis.

### 2.5 Handling of missing data

Since the UK Biobank collected random blood samples during the baseline recruitment, only 6% of the total participants provided fasting blood samples, defined as blood samples taken after at least 7 h of fasting for this study. For those who did not provide a fasting blood sample, a case of high fasting glucose level was defined for individuals taking medication to manage high fasting glucose levels, while those not taking medication were treated as missing since confirmation of their fasting levels was not feasible. This same approach was applied when identifying cases for high triglycerides.

In cases where participants did not provide data on medication use, a case of high fasting glucose levels was established if fasting glucose levels exceeded the threshold specified in Table S1. Conversely, the rest were treated as missing, considering uncertainty regarding whether observed fasting glucose levels were influenced by medication use. Similar logic was extended to ascertain cases for high triglycerides, high blood pressure, and low HDL‐cholesterol levels.

### 2.6 Measurement of confounding variables

Socioeconomic position was characterized using the Townsend Deprivation index.^16^ Self‐reported smoking status, alcohol intake frequency, and highest qualification attained were subsequently recoded as follows: smoking status (never/previous/current), alcohol intake frequency (monthly or less/weekly/daily), and highest qualification obtained (below General Certificate of Secondary Education [GCSE], GCSE, A‐level, or degree holder). Physical activity level was evaluated using the validated International Physical Activity Questionnaire (IPAQ) and categorized as low, moderate, or high based on IPAQ research committee guidelines.^17^ Intakes of fruit, vegetable, and tea were self‐reported by the participants. Fruit intake was calculated by summing the intake of fresh and dried fruit while vegetable intake was calculated by summing the cooked and raw vegetable intake. Tea intake was measured in cups per day.

### 2.7 Mendelian randomization analysis

To complement the observational analysis, a two‐sample Mendelian randomization study was undertaken to estimate the causal effect of habitual coffee intake on metabolic syndrome risk. This instrumental variable analysis is less susceptible to residual confounding and reverse causation and relies on three instrumental variable assumptions.^13^ First, the instruments should be strongly associated with the exposure of interest. Second, there should be no unmeasured confounding of instruments on outcome. Third, the instruments should be independent of the outcome given the exposure and the confounders.

Single‐nucleotide polymorphisms (SNPs) associated with coffee consumption were obtained from a large genome‐wide association study conducted by the Coffee and Caffeine Genetics Consortium, which did not include data from the UK Biobank.^18^ These included (i) coffee consumption in cups per day among coffee drinkers (phenotype 1, *n* = 129 488, of which 93.9% were Europeans) and (ii) no/low versus high coffee consumption (phenotype 2, *n* = 65 842, of which 98.2% were Europeans). Only independent SNPs (*r* ^*2*^ < 0.001) that achieved genome‐wide significance (*p <* 5 × 10^−8^) in the trans‐ethnic meta‐analysis were selected (Table S3).

Genetic associations between the identified SNPs for cups of coffee consumed (phenotype 1) and being a coffee drinker (phenotype 2) and metabolic syndromes and its components, including high fasting glucose, high triglycerides, central obesity, high blood pressure, and low HDL, were derived using individual‐level data in the UK Biobank. Effect estimates were obtained using multivariable logistic regression. Adjustments were made for age, sex, top 20 principal components, genotype array, and assessment center. Exclusions encompassed participants with subpar genotyping quality (missing rate > 0.01), ≥10 putative third‐degree relatives, sex chromosome aneuploidy, mismatch between reported and genetic sex, and inconsistency in reported and genetic ethnicity. Genetic associations involving outcomes were observed among up to 360 903 participants.

### 2.8 Statistical analyses

All analyses were done using R version 4.1.1.^19^ mendelian randomization (MR) analyses were performed using the “*TwoSampleMR*” package^20^, ^21^ and “*MVMR*” package.^22^ To address the issue of multiple testing, we established a *p*‐value threshold for statistical significance across all analyses at 0.0083 (derived from dividing 0.05 by the six distinct outcomes examined). All scripts used for data cleaning and analyses were included in the Supporting Information.

We employed multivariable logistic regression to scrutinize the cross‐sectional associations between coffee consumption and metabolic syndrome, along with its constituent components. In this analysis, individuals who did not consume coffee served as the reference group. Effect estimates were meticulously adjusted for potential confounding variables, including age, sex, Townsend deprivation index, highest educational qualification achieved, smoking status, frequency of alcohol intake, level of physical activity, and consumption of fruits, vegetables, and tea. Furthermore, when evaluating data derived from 24‐h recalls, we introduced coffee type as a covariate. To explore the interplay between the use of milk, sugar, and artificial sweeteners with coffee and their collective impact on outcomes, we undertook subanalyses stratified by these factors whenever feasible. Specifically, for instant and filtered coffee, we conducted stratification based on milk use, and for all coffee types except espresso, we executed stratification based on the use of sugar and artificial sweeteners.

We calculated the *R* ^*2*^ (only phenotype 1) and *F* statistics of the instruments, serving as indicators of instrument strength. These calculations adhered to the methodologies outlined in a prior Mendelian randomization study,^23^ where a higher *F* statistic indicated diminished evidence of weak instrument bias. Moreover, we harmonized the effect alleles of all instruments with the allele associated with increased coffee consumption. To assess the causal link between coffee consumption and metabolic syndrome, in addition to its constituent conditions, we employed the inverse variance weighted (IVW) method, with multiplicative random effect models. The IVW estimator operates under the assumption of balanced horizontal pleiotropy.^24^ Notably, a high level of heterogeneity within instrument‐specific Wald Ratios, as determined by Cochran's *Q* test, may suggest the potential inclusion of invalid instruments. To fortify the robustness of our findings, a series of sensitivity analyses were conducted, grounded in diverse assumptions. The MR‐Egger estimator, designed to account for overall directional pleiotropic effects, was employed. It assumes that the instrument's strength is independent of the direct effect (InSIDE assumption). The presence of a statistically significant MR‐Egger intercept (*p* < 0.05) signifies the existence of a horizontal pleiotropic effect.^24^ Additionally, the weighted median method, predicated on at least 50% of the weight arising from valid SNPs, was employed.^25^

Since smoking status and alcohol consumption were well‐recognized confounding factors in the associations between coffee consumption and various health outcomes^26^ and genetic predictors of coffee consumption were found to be associated with both traits as well,^26^ we conducted multivariable MR to obtain effect estimates adjusted for alcohol consumption and liability to smoking initiation. Genetic variants linked to alcohol consumption and smoking initiation liabilities were identified with genome‐wide significance (*p <* 5 × 10^−8^) from the genome‐wide association study (GWAS) and Sequencing Consortium of Alcohol and Nicotine use study,^27^ featuring participants exclusively of European ancestry, distinct from those in the UK Biobank cohort (*n* = 249 171 for smoking initiation; *n* = 226 223 for alcohol consumption). For the multivariable MR analysis, genetic predictors of coffee consumption were based on stage one results from the CCGC meta‐analysis, limited to European participants. The lists of SNPs used in the multivariable MR for both phenotypes are provided in Tables S4 and S5. We excluded SNPs in linkage disequilibrium with other instruments using a more lenient cutoff (*r* ^2^ < 0.1) so that we have a sufficient number of instruments and maintain statistical power for multivariable MR. Genetic associations for exposure and outcome were aligned to the same effect allele, and additionally effect allele frequency for palindromic SNPs. In cases where the target SNPs were absent from any dataset, proxy SNPs (*r* ^*2*^ ≥ 0.8) were used. The causal effect estimates of coffee consumption on metabolic syndrome and its individual components were subsequently computed through multivariable IVW and multivariable MR‐Egger approaches.^28^, ^29^

### 2.9 Use of large language models

During the preparation of this work, the authors used ChatGPT 3.5 to improve the flow, clarity, coherence, and grammar of the text drafted by the authors. After using this tool/service, the senior author (JCYL) reviewed and edited the content as needed and takes full responsibility for the content of the publication.

## 3 RESULTS

### 3.1 Participant characteristics

**FIGURE 1:**
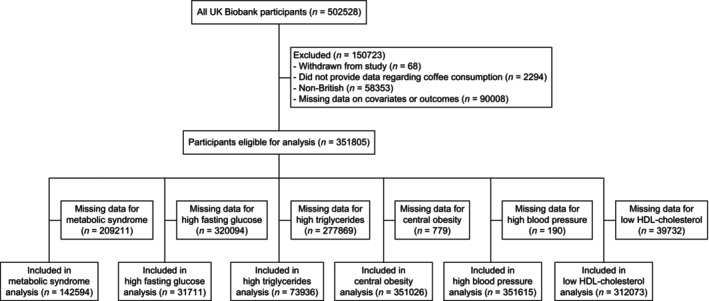
Participant exclusion flowchart for the cross‐sectional analysis.

After excluding individuals who withdrew consent, were non‐British, lacked coffee intake data, or had missing covariate data, 351 805 participants were included (Figure 1). Table 1 displays participant characteristics by daily coffee consumption. Coffee drinkers tended to have a higher proportion of males, smokers, frequent alcohol consumption, education attainment, and lower tea intake compared to nondrinkers.

**TABLE 1:** Characteristics of participants included in cross‐sectional analysis (*n* = 351 805).

Variables	Coffee consumption (cups/day)							
	0	<1	1	2	3	4	5	6 or more
Participants, *n*	72 860	24 309	71 116	67 516	44 629	30 940	18 260	22 175
Age, years	55.5 (8.2)	56.4 (7.9)	57.3 (8.0)	57.2 (7.9)	57.0 (7.9)	56.6 (7.9)	56.0 (8.0)	55.6 (8.0)
Male, %	43.5	47.0	45.2	48.1	49.9	52.2	54.2	55.6
Smoking status, %								
Never	57.6	58.3	57.4	55.7	54.3	50.9	47.2	39.9
Previous	33.2	34.2	35.7	36.1	36.6	37.4	37.2	35.9
Current	9.2	7.5	6.9	8.2	9.0	11.7	15.6	24.1
Alcohol consumption frequency, %								
Monthly or less	37.7	27.1	24.9	21.8	21.6	22.7	25.8	32.3
Weekly	46.0	52.7	53.1	53.2	52.5	52.4	51.1	46.9
Daily	16.3	20.2	22.0	25.0	25.8	24.8	23.1	20.8
Fruit intake, tbsp	3.0 (2.5)	3.1 (2.4)	3.2 (2.5)	3.2 (2.5)	3.0 (2.4)	2.9 (2.3)	2.8 (2.4)	2.7 (2.6)
Vegetable intake, tbsp	4.7 (3.3)	4.7 (3.0)	4.9 (3.0)	4.9 (3.0)	4.8 (3.0)	4.8 (3.1)	4.7 (3.1)	4.8 (3.5)
Tea intake, cups/day	4.7 (3.3)	4.6 (2.8)	4.0 (2.5)	3.3 (2.4)	2.6 (2.2)	2.2 (2.3)	1.8 (2.5)	1.9 (3.3)
IPAQ category, %								
Low	20.0	19.3	17.0	17.1	18.3	19.2	21.4	22.3
Moderate	39.0	42.0	41.6	42.1	42.1	41.3	39.6	38.1
High	41.0	38.7	41.4	40.8	39.6	39.5	39.0	39.6
Highest qualification obtained, %								
Below GCSE	26.1	18.2	21.3	18.6	18.1	20.2	20.6	23.9
GCSE	24.7	21.8	21.5	21.3	20.9	22.0	22.8	24.1
Advanced level	7.7	8.4	7.6	7.7	7.7	7.4	7.3	7.6
Degree holder	41.6	51.6	49.7	52.4	53.3	50.4	49.4	44.5

*Note*: Values are mean (SD) for age and percentages for count variables. Coffee consumption was analyzed as a categorical variable.

Abbreviations: GCSE, general certificate of secondary education; IPAQ, International Physical Activity Questionnaire.

### 3.2 Coffee intake and metabolic syndrome

**FIGURE 2:**
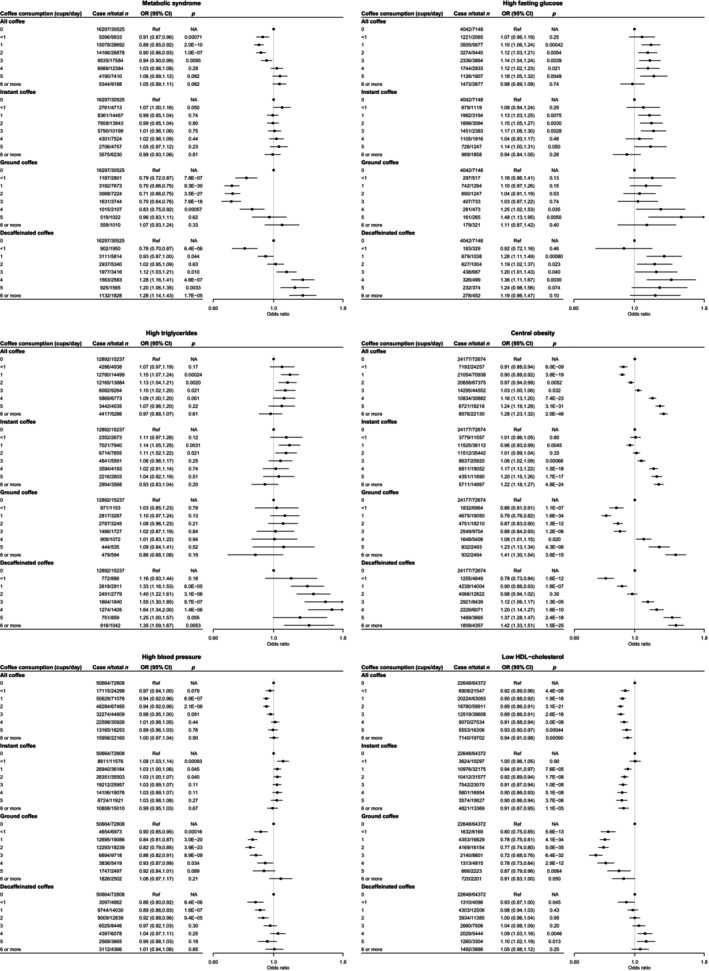
Associations between overall coffee consumption, as well as stratified by coffee types, and metabolic syndrome, high fasting glucose, high triglycerides, central obesity, high blood pressure, and low HDL‐cholesterol. Error bars are 95% CI. All data used were from the baseline food frequency questionnaire. All effect estimates were adjusted for age, sex, smoking status, alcohol consumption frequency, highest qualification obtained, vegetable intake, fruit intake, tea intake, and physical activity level. CI, confidence interval.

Figure 2 depicts the association between daily coffee intake and metabolic syndrome prevalence among 142 594 participants after adjustment for sociodemographic and lifestyle factors. Consuming up to three cups per day was associated with lower metabolic syndrome odds compared with abstainers (1 cup/day odds ratio [OR]: 0.88, 95% confidence interval [CI]: 0.85–0.92; 2 cups/day OR: 0.90, 95% CI: 0.86–0.93; 3 cups/day OR: 0.94, 95% CI: 0.90–0.99). However, intakes of four cups or more daily were potentially associated with higher metabolic syndrome odds (4 cups/day OR: 1.03, 95% CI: 0.98–1.08; 5 cups/day OR: 1.06, 95% CI: 0.99–1.12). Coffee intake displayed positive associations with high fasting glucose and triglycerides, while inverse associations were observed for high blood pressure and low HDL‐cholesterol. For central obesity, a J‐shaped relationship was found (Figure 2).

**FIGURE 3:**
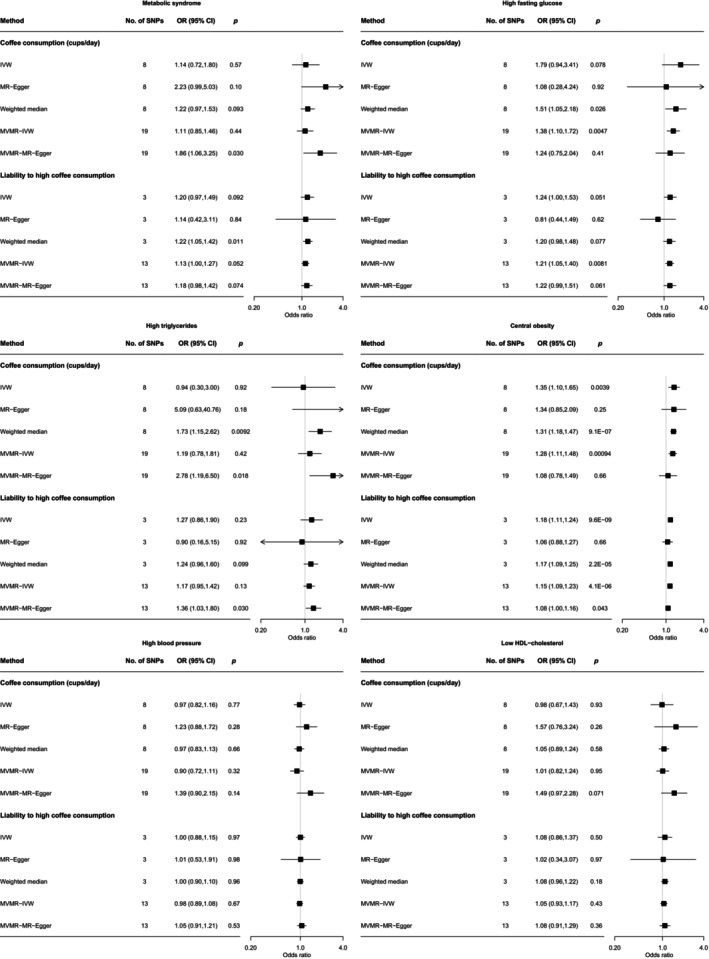
Results of MR analysis between coffee intake and metabolic syndrome, high fasting glucose, high triglycerides, central obesity, high blood pressure, and low HDL‐cholesterol. Error bars are 95% CI. Effect estimates of MVMR are adjusted for alcohol consumption and liability to smoke initiation. Coffee consumption was measured among coffee consumers only. For liability to high coffee consumption, high coffee consumption was defined as four cups or more per day. CI, confidence interval; MVMR, multivariable mendelian randomization.

We identified eight instruments for phenotype 1 (cups of coffee consumed, *R* ^2^: 0.4%, range of *F* statistics: 9.0–196.0) and three instruments for phenotype 2 (liability to high coffee consumption, range of *F* statistics: 44.4–100.0) for the MR analysis (Figure S1). Results did not support a causal effect of genetically predicted coffee intake on metabolic syndrome risk (Figure 3 and Tables S6 and S7). Despite heterogeneous instruments based on Cochran's *Q* test, MR‐Egger regression suggested did not provide strong evidence for overall horizontal pleiotropy. Additional multivariable Mendelian randomization adjusting for smoking and alcohol attenuation yielded consistent results (Figure S2 and Tables S8 and S9). However, coffee intake was causally associated with higher central obesity risk based on analyses with both phenotypes. There was also suggestive evidence for a causal effect on high fasting glucose (Figure 3).

### 3.3 Coffee type, additives, and metabolic outcomes

Stratified analyses by coffee subtype aligned mostly with the overall findings, with some exceptions (Figure 2). For example, instant coffee intake displayed null associations with metabolic syndrome and high blood pressure, while decaffeinated coffee was not associated with low HDL‐cholesterol. Intakes of latte/cappuccino were inversely linked to metabolic syndrome and high blood pressure only, whereas espresso intake showed no associations (Figures S3–S6). Regarding additives, milk and sugar did not substantially modify the associations. However, artificial sweetener use was positively associated with all outcomes among instant coffee drinkers. Similarly, filtered coffee with sweeteners displayed positive associations except for glucose and triglycerides. Avoiding sweeteners was associated with lower metabolic syndrome, central obesity, and low HDL‐cholesterol among filtered and latte/cappuccino consumers.

## 4 DISCUSSION

In this large population‐based study of over 350 000 UK Biobank participants, we observed that low‐to‐moderate habitual coffee consumption (1–3 cups per day) was associated with lower odds of prevalent metabolic syndrome in cross‐sectional analyses. However, these observational relationships were not corroborated by our two‐sample Mendelian randomization study, which suggested the associations are likely susceptible to residual confounding, reverse causation, or bias. On the other hand, both high self‐reported intake and genetically predicted elevated coffee consumption appeared to be associated with increased risk of central obesity. We also demonstrated for the first time in a European population that the use of artificial sweeteners in coffee was linked to higher odds of metabolic syndrome and its components, highlighting the potential importance of coffee additives.

The null causal associations between coffee intake and metabolic syndrome align with earlier Mendelian randomization conducted in a Danish cohort, which similarly found protective observational associations that were not affirmed by genetic analyses.^30^ By using data from separate, nonoverlapping studies for exposure and outcome, and overall larger sample sizes, our study has more statistical power than the previous study.^13^ Furthermore, consistent results were observed in multiple sensitivity analyses and after accounting for potential pleiotropic effects in the multivariable Mendelian randomization analysis. Taken together, these studies imply the inverse relationships reported frequently in cross‐sectional studies^31^, ^32^ may be largely ascribed to confounding lifestyle and socioeconomic factors that correlate with coffee drinking,^33^ rather than a true protective metabolic effect of coffee itself. Our supplemental observational analysis by coffee subtype provides further evidence of confounding, as the apparently beneficial associations with metabolic syndrome were only seen among consumers of ground and decaffeinated coffee, but not instant coffee. Residual confounding by a healthy diet and lifestyle factors that associate more strongly with filtered and decaffeinated coffee consumption likely explains these discrepant findings.^33^

In contrast, for central obesity, we demonstrated directionally concordant relationships for both self‐reported intake and genetically predicted coffee consumption, including in sensitivity analyses less susceptible to pleiotropy. This aligns with an earlier Mendelian randomization study that reported causal associations between coffee and elevated obesity risk^34^ that observed a positive association between coffee consumption and obesity, but contradicts observational studies linking coffee to lower obesity risk,^35^ warranting further mechanistic research.

While caffeine may increase thermogenesis,^36^ and coffee polyphenols exhibit antiobesity properties,^37^ psychological pathways could be relevant. Observational data supports positive associations between caffeine intake, psychological stress,^38^, ^39^ and obesity.^40^ A recent two‐sample MR study by Larsson et al. reported genetically predicted higher plasma caffeine levels were associated with lower BMI, fat mass, and type 2 diabetes risk. Nonetheless, while cortisol, a marker of stress, is not causally linked to adiposity,^41^ stress elicits other biological responses such as increase in inflammatory cytokines.^42^ Further research should investigate if stress mediates links between coffee and central obesity.

Additionally, we demonstrated for the first time in a European ancestry population that the use of artificial sweeteners in coffee was associated with higher odds of metabolic syndrome and its cardiometabolic components. These findings align with prior observational studies linking routine artificial sweetener consumption to deleterious effects on weight management, glycemic control, and metabolic health.^43^ Proposed mechanisms for the observed associations include artificial sweetener modulation of taste preferences and food cravings through interference in learned caloric associations.^44^ Regular exposure to nonnutritive sweeteners may distort the innate relationship between sweet taste perception and energy homeostasis, promoting excess food intake and subsequent metabolic dysregulation. Further, artificially sweetened beverages have been shown in human studies to induce glucose intolerance through alterations in gut microbiota composition and function.^45^ Nonetheless, since the evidence regarding the effect of artificial sweetener use on weight management and glycemic control was highly mixed,^46^, ^47^ reverse causation remains an alternative explanation if participants with underlying metabolic conditions preferentially added artificial sweeteners to coffee due to health concerns. Additional longitudinal studies and clinical trials are warranted to further evaluate the potential metabolic effects of coffee additives like artificial sweeteners.

Major strengths of our study include the large sample size, detailed characterization of coffee intake, and the multifaceted analytical approach incorporating both conventional multivariable regression and Mendelian randomization methods. Limitations should also be acknowledged. First, the reliance on nonfasting blood samples for defining metabolic syndrome components in a majority of participants may have introduced misclassification bias. However, we leveraged medication use data to improve case ascertainment. Second, stratification by use of specific coffee additives yielded reduced sample sizes and limited power for certain analyses. Third, the genetic instruments employed may have been influenced by pleiotropy, as they were associated with potential confounders like smoking, alcohol use, and tea drinking based on published evidence.^26^ However, sensitivity analyses using methods more robust to pleiotropy yielded consistent inferences. Fourth, the use of a dichotomized coffee intake phenotype in Mendelian randomization may have violated instrumental variable assumptions.^48^ Finally, the UK Biobank cohort comprises predominantly White British individuals, limiting generalizability of findings to other ancestral populations.

In conclusion, while low‐moderate coffee consumption demonstrated inverse associations with metabolic syndrome cross‐sectionally, our genetic analyses do not provide evidence to support causal protective effects on metabolic risk. However, higher coffee intake may contribute to elevated central obesity risk. We also observed heterogeneity by coffee subtype and additives like artificial sweeteners, which may importantly modify metabolic associations. Additional longitudinal cohort studies and randomized trials with detailed coffee phenotyping, expanded coffee‐related genetic data, and diverse study populations are warranted to clarify the impact of coffee drinking on cardiometabolic health. Elucidating the complex relationship among coffee, additives, and metabolic outcomes can help inform evidence‐based dietary recommendations.

## AUTHOR CONTRIBUTIONS

Jimmy Chun Yu Louie and Tommy Hon Ting Wong designed research. Shan Luo, Shiu Lun Au Yeung, and Jimmy Chun Yu Louie provided essential materials. Tommy Hon Ting Wong analyzed the data, performed statistical analysis, and drafted the paper. All authors critically evaluated and amended the manuscript. Tommy Hon Ting Wong and Jimmy Chun Yu Louie had primary responsibility for the final content. All authors read and approved the final manuscript.

## FUNDING INFORMATION

This study was supported by an internal grant from the Faculty of Science, The University of Hong Kong.

## CONFLICT OF INTEREST STATEMENT

The authors declare that they have no conflict of interest.

## Supporting information


**Data S1.** Supporting Information.

**Data S2.** Supporting Information.

## Data Availability

The data that support the findings of this study are available from the UK Biobank, but restrictions apply to the availability of these data, which were used under license for the current study, and so are not publicly available.
