# Supplementary material for: Association between coffee consumption and metabolic syndrome: A cross‐sectional and Mendelian randomization study
Source: J Diabetes. 2024 Oct 10;16(10):e70004. doi: 10.1111/1753-0407.70004 (PMC11467012; doi:10.1111/1753-0407.70004)
Supplement: Supplementary file 1 — Data S1. Supporting Information. [file JDB-16-e70004-s002.pdf]

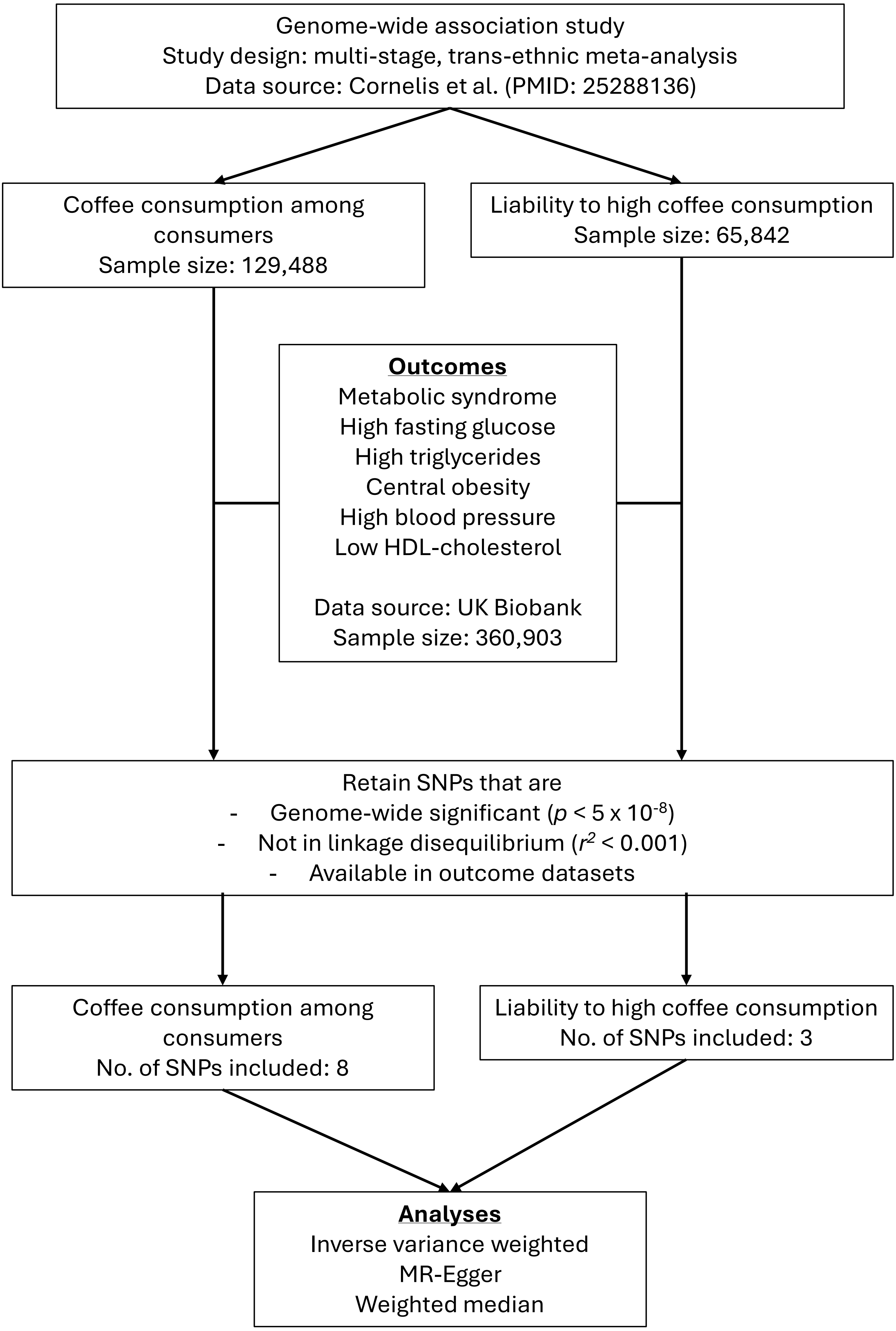

Supplemental figure 1 – Study flow chart of mendelian randomization analysis. SNP, single nucleotide polymorphisms.

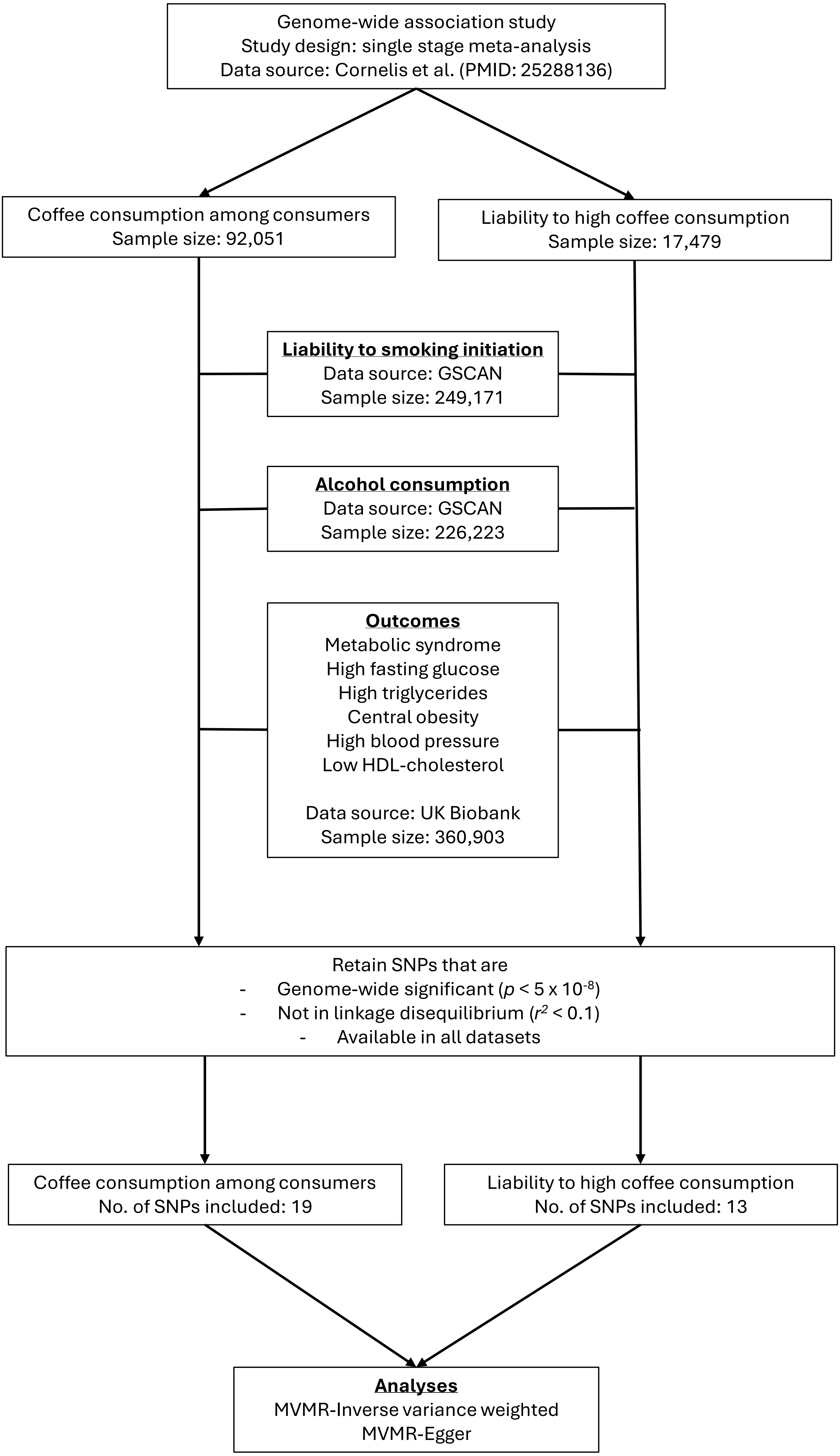

Supplemental figure 2 – Study flow chart of multivariable mendelian randomization (MVMR) analysis. SNP, single nucleotide polymorphisms.

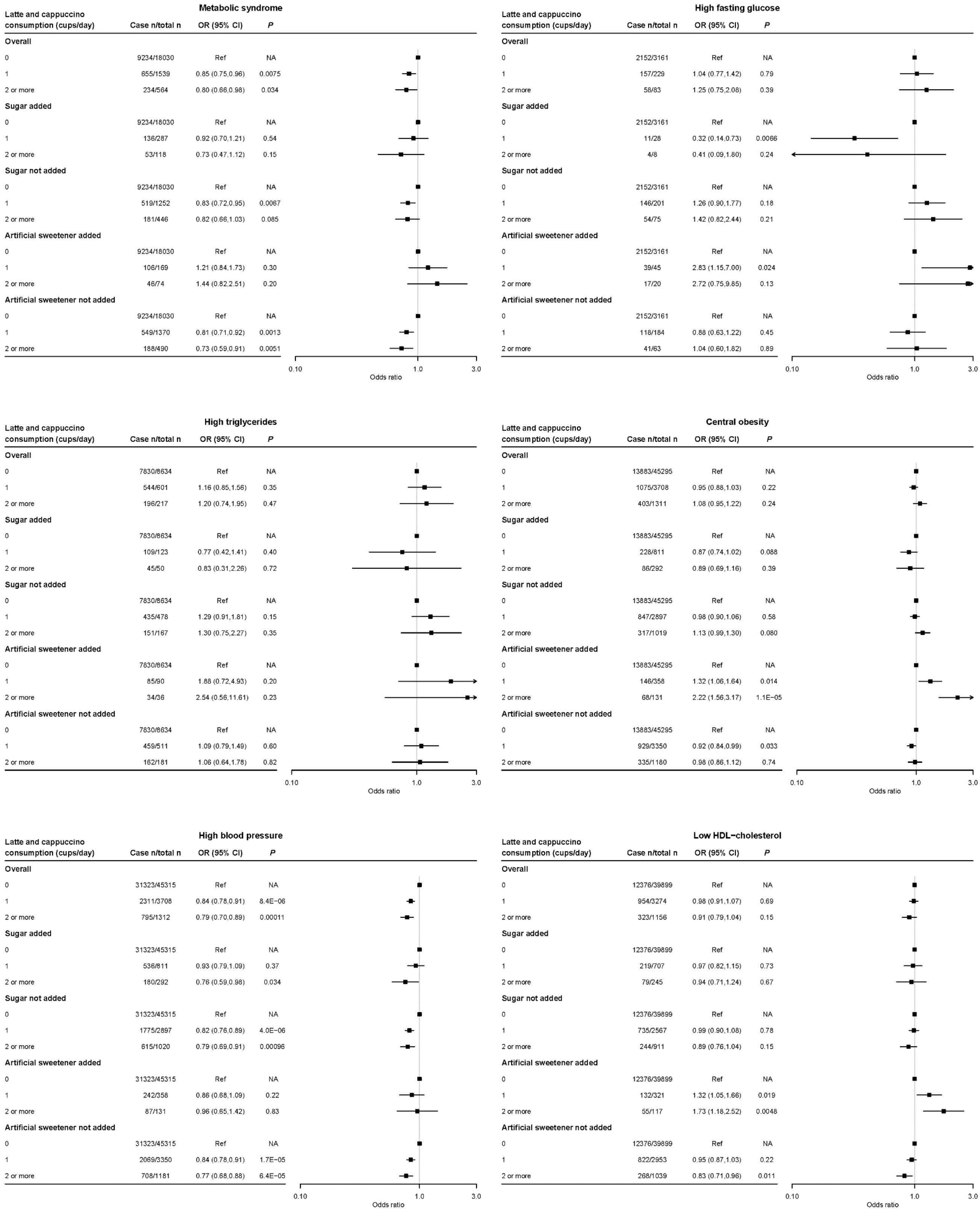

Supplemental figure 3 – Association between latte and cappuccino consumption and all outcomes, stratified by the use of milk, sugar, and artificial sweetener. All data were from 24-hour recalls of UK Biobank. All effect estimates were adjusted for age, sex, smoking status, alcohol consumption frequency, vegetable intake, fruit intake, tea intake, physical activity level, and highest qualification obtained. Error bars depict 95% CI.

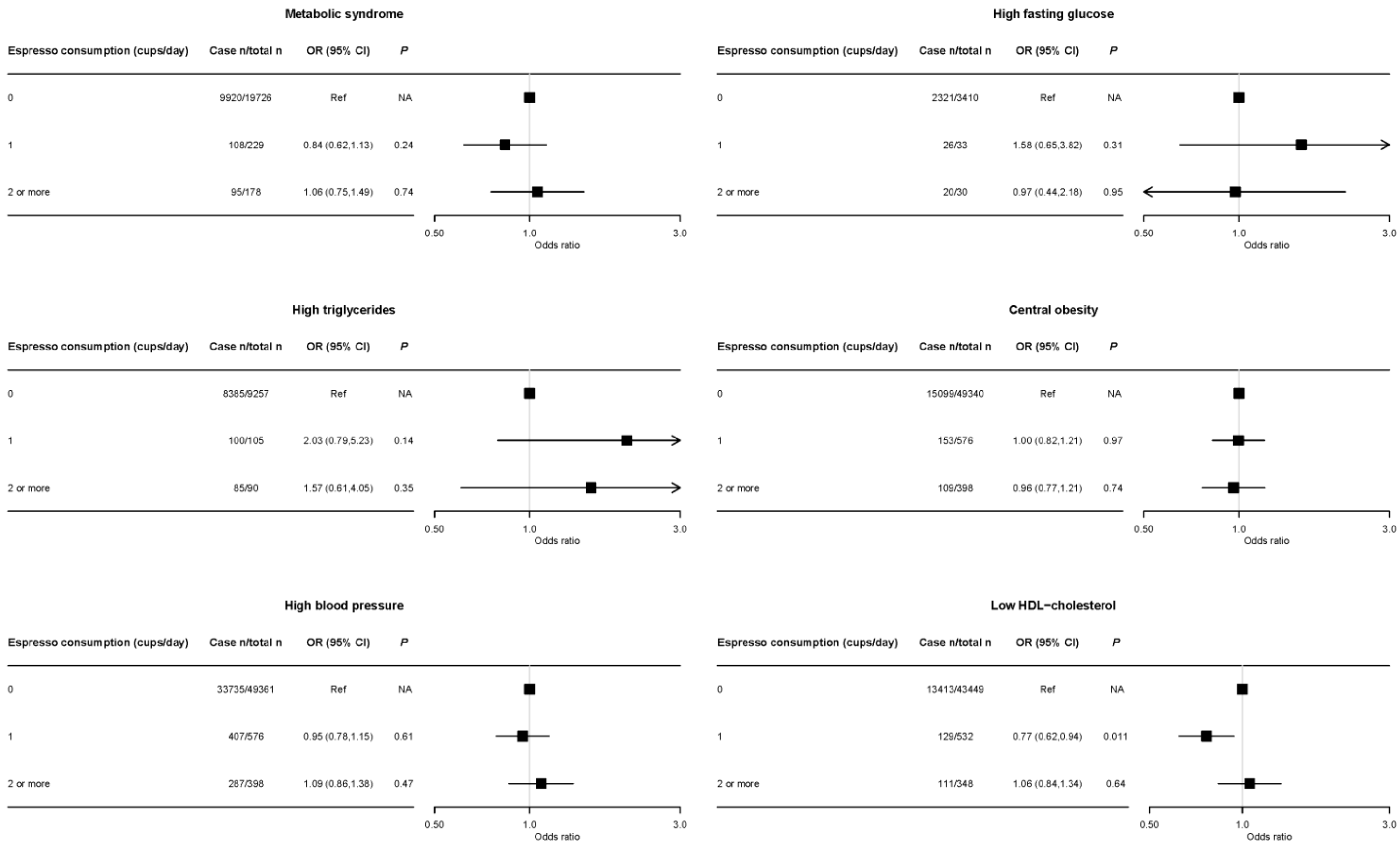

Supplemental figure 4 – Association between espresso consumption and all outcomes. All data were from 24-hr recalls of UK Biobank. All effect estimates were adjusted for age, sex, smoking status, alcohol consumption frequency, vegetable intake, fruit intake, tea intake, physical activity level, and highest qualification obtained. Error bars depict 95% CI.

Metabolic syndrome

Instant coffee consumption (cups/day)	Case n/total n	OR (95% CI)	P
**Milk added**			
0	4718/10171	Ref	NA
1	1377/2735	0.97 (0.88,1.07)	0.53
2	1243/2366	1.07 (0.96,1.19)	0.22
3 or more	1803/3213	1.17 (1.06,1.30)	0.0017
**Milk not added**			
0	4718/10171	Ref	NA
1	296/472	1.55 (1.24,1.93)	0.00010
2	251/441	1.29 (1.03,1.62)	0.026
3 or more	435/735	1.43 (1.19,1.71)	0.00016
**Sugar added**			
0	4718/10171	Ref	NA
1	415/763	0.89 (0.75,1.06)	0.21
2	313/614	0.78 (0.64,0.94)	0.011
3 or more	445/779	0.97 (0.81,1.16)	0.71
**Sugar not added**			
0	4718/10171	Ref	NA
1	1258/2444	1.09 (0.98,1.21)	0.11
2	1181/2193	1.22 (1.10,1.37)	0.00036
3 or more	1793/3169	1.30 (1.17,1.43)	4.6E−07
**Artificial sweetener added**			
0	4718/10171	Ref	NA
1	272/378	2.05 (1.57,2.68)	1.6E−07
2	236/324	2.16 (1.61,2.90)	2.9E−07
3 or more	409/571	2.16 (1.73,2.70)	1.1E−11
**Artificial sweetener not added**			
0	4718/10171	Ref	NA
1	1401/2829	0.96 (0.87,1.06)	0.41
2	1258/2483	1.02 (0.92,1.14)	0.66
3 or more	1829/3377	1.12 (1.02,1.23)	0.024

High fasting glucose

Instant coffee consumption (cups/day)	Case n/total n	OR (95% CI)	P
**Milk added**			
0	1103/1682	Ref	NA
1	311/454	1.05 (0.83,1.34)	0.69
2	284/392	1.30 (1.00,1.69)	0.053
3 or more	433/612	1.29 (1.02,1.62)	0.030
**Milk not added**			
0	1103/1682	Ref	NA
1	70/93	1.53 (0.91,2.56)	0.11
2	55/83	1.03 (0.62,1.70)	0.92
3 or more	111/157	1.30 (0.88,1.93)	0.19
**Sugar added**			
0	1103/1682	Ref	NA
1	53/97	0.60 (0.39,0.93)	0.023
2	29/63	0.42 (0.24,0.73)	0.0020
3 or more	39/93	0.34 (0.21,0.54)	5.2E−06
**Sugar not added**			
0	1103/1682	Ref	NA
1	328/450	1.31 (1.02,1.68)	0.034
2	310/412	1.53 (1.17,2.00)	0.0017
3 or more	505/676	1.63 (1.30,2.04)	2.5E−05
**Artificial sweetener added**			
0	1103/1682	Ref	NA
1	92/112	1.95 (1.15,3.29)	0.013
2	84/101	1.99 (1.14,3.49)	0.016
3 or more	136/167	2.26 (1.45,3.50)	0.00030
**Artificial sweetener not added**			
0	1103/1682	Ref	NA
1	289/435	0.98 (0.77,1.25)	0.89
2	255/374	1.13 (0.87,1.47)	0.35
3 or more	408/602	1.15 (0.92,1.44)	0.22

High triglycerides

Instant coffee consumption (cups/day)	Case n/total n	OR (95% CI)	P
**Milk added**			
0	3957/4419	Ref	NA
1	1206/1316	1.15 (0.91,1.46)	0.25
2	1079/1167	1.35 (1.04,1.75)	0.023
3 or more	1487/1626	1.39 (1.11,1.75)	0.0046
**Milk not added**			
0	3957/4419	Ref	NA
1	254/275	1.40 (0.85,2.28)	0.18
2	221/247	1.09 (0.69,1.72)	0.72
3 or more	366/402	1.41 (0.95,2.10)	0.087
**Sugar added**			
0	3957/4419	Ref	NA
1	376/407	1.12 (0.74,1.69)	0.58
2	276/301	1.05 (0.66,1.65)	0.85
3 or more	373/411	1.05 (0.71,1.55)	0.82
**Sugar not added**			
0	3957/4419	Ref	NA
1	1084/1184	1.24 (0.97,1.58)	0.093
2	1024/1113	1.41 (1.08,1.83)	0.010
3 or more	1480/1617	1.55 (1.23,1.95)	0.00019
**Artificial sweetener added**			
0	3957/4419	Ref	NA
1	229/243	1.58 (0.87,2.86)	0.13
2	207/222	1.43 (0.80,2.53)	0.22
3 or more	341/357	3.02 (1.75,5.22)	7.7E−05
**Artificial sweetener not added**			
0	3957/4419	Ref	NA
1	1231/1348	1.14 (0.90,1.43)	0.27
2	1093/1192	1.26 (0.99,1.62)	0.064
3 or more	1512/1671	1.23 (0.99,1.53)	0.062

Central obesity

Instant coffee consumption (cups/day)	Case n/total n	OR (95% CI)	P
**Milk added**			
0	7172/25021	Ref	NA
1	2000/7114	0.94 (0.88,1.00)	0.044
2	1860/5999	1.07 (1.01,1.14)	0.035
3 or more	2828/7967	1.25 (1.18,1.33)	1.4E−13
**Milk not added**			
0	7172/25021	Ref	NA
1	406/1217	1.23 (1.08,1.40)	0.0013
2	398/1119	1.35 (1.19,1.54)	6.0E−06
3 or more	697/1877	1.34 (1.21,1.49)	4.9E−08
**Sugar added**			
0	7172/25021	Ref	NA
1	528/2049	0.78 (0.70,0.87)	6.0E−06
2	460/1619	0.89 (0.79,1.00)	0.046
3 or more	637/2005	1.03 (0.92,1.14)	0.62
**Sugar not added**			
0	7172/25021	Ref	NA
1	1878/6282	1.05 (0.99,1.12)	0.12
2	1798/5499	1.19 (1.11,1.27)	4.3E−07
3 or more	2888/7839	1.33 (1.25,1.41)	8.4E−21
**Artificial sweetener added**			
0	7172/25021	Ref	NA
1	371/808	1.79 (1.54,2.07)	7.4E−15
2	348/746	1.85 (1.59,2.16)	2.9E−15
3 or more	564/1222	1.73 (1.53,1.96)	4.7E−18
**Artificial sweetener not added**			
0	7172/25021	Ref	NA
1	2035/7523	0.91 (0.85,0.96)	0.0016
2	1910/6372	1.04 (0.98,1.11)	0.21
3 or more	2961/8622	1.21 (1.14,1.29)	1.1E−10

High blood pressure

Instant coffee consumption (cups/day)	Case n/total n	OR (95% CI)	P
**Milk added**			
0	16570/25033	Ref	NA
1	5010/7120	1.04 (0.98,1.11)	0.21
2	4195/5997	1.02 (0.96,1.09)	0.53
3 or more	5580/7969	1.05 (0.99,1.12)	0.11
**Milk not added**			
0	16570/25033	Ref	NA
1	901/1217	1.19 (1.03,1.37)	0.016
2	802/1119	1.13 (0.98,1.30)	0.094
3 or more	1371/1880	1.23 (1.09,1.38)	0.00056
**Sugar added**			
0	16570/25033	Ref	NA
1	1485/2049	1.00 (0.89,1.11)	0.94
2	1158/1619	0.95 (0.84,1.07)	0.38
3 or more	1429/2008	0.99 (0.88,1.10)	0.80
**Sugar not added**			
0	16570/25033	Ref	NA
1	4426/6288	1.08 (1.01,1.15)	0.018
2	3839/5497	1.06 (0.99,1.14)	0.076
3 or more	5522/7841	1.11 (1.04,1.18)	0.0014
**Artificial sweetener added**			
0	16570/25033	Ref	NA
1	621/808	1.35 (1.13,1.61)	0.00094
2	554/745	1.24 (1.03,1.48)	0.020
3 or more	895/1221	1.19 (1.03,1.37)	0.018
**Artificial sweetener not added**			
0	16570/25033	Ref	NA
1	5290/7529	1.04 (0.98,1.10)	0.23
2	4443/6371	1.02 (0.95,1.09)	0.58
3 or more	6056/8628	1.07 (1.00,1.14)	0.034

Low HDL−cholesterol

Instant coffee consumption (cups/day)	Case n/total n	OR (95% CI)	P
**Milk added**			
0	6427/21990	Ref	NA
1	1890/6261	0.96 (0.90,1.03)	0.26
2	1665/5314	1.00 (0.93,1.07)	0.98
3 or more	2430/7075	1.07 (1.00,1.14)	0.038
**Milk not added**			
0	6427/21990	Ref	NA
1	368/1070	1.16 (1.02,1.33)	0.026
2	322/981	1.10 (0.96,1.27)	0.18
3 or more	551/1638	1.02 (0.91,1.15)	0.71
**Sugar added**			
0	6427/21990	Ref	NA
1	589/1812	0.95 (0.86,1.06)	0.39
2	456/1452	0.87 (0.77,0.98)	0.027
3 or more	655/1791	1.05 (0.94,1.17)	0.38
**Sugar not added**			
0	6427/21990	Ref	NA
1	1669/5519	1.01 (0.94,1.08)	0.85
2	1531/4843	1.07 (0.99,1.15)	0.071
3 or more	2326/6922	1.06 (1.00,1.14)	0.062
**Artificial sweetener added**			
0	6427/21990	Ref	NA
1	315/718	1.50 (1.28,1.75)	4.6E−07
2	282/678	1.38 (1.17,1.63)	0.00010
3 or more	491/1097	1.44 (1.26,1.64)	7.2E−08
**Artificial sweetener not added**			
0	6427/21990	Ref	NA
1	1943/6613	0.94 (0.89,1.01)	0.071
2	1705/5617	0.98 (0.91,1.05)	0.52
3 or more	2490/7616	1.02 (0.96,1.08)	0.59

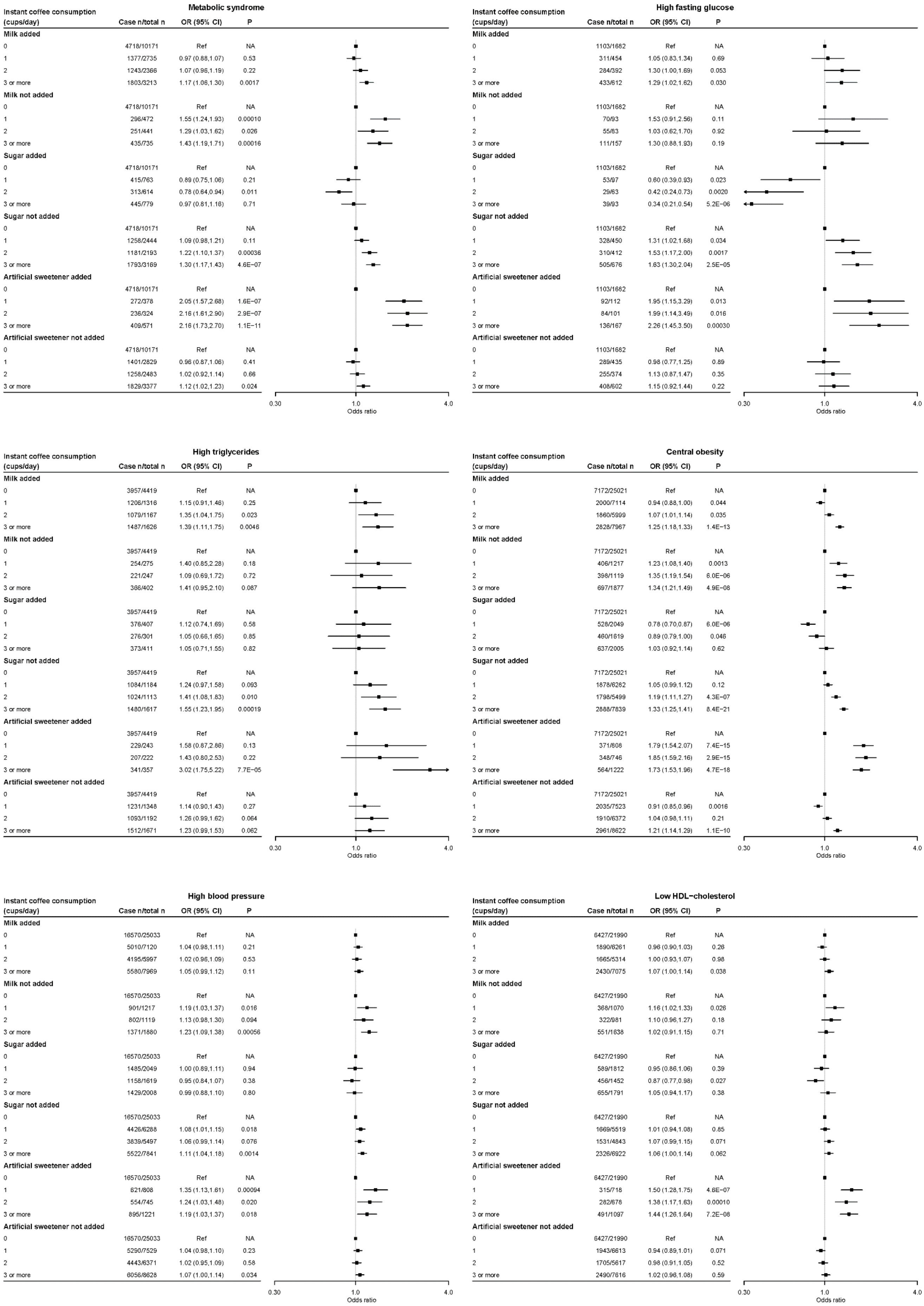

Supplemental figure 5 – Association between instant coffee consumption and all outcomes, stratified by the use of milk, sugar, and artificial sweetener. All data were from 24-hour recalls of UK Biobank. All effect estimates were adjusted for age, sex, smoking status, alcohol consumption frequency, vegetable intake, fruit intake, tea intake, physical activity level, and highest qualification obtained. Error bars depict 95% CI.

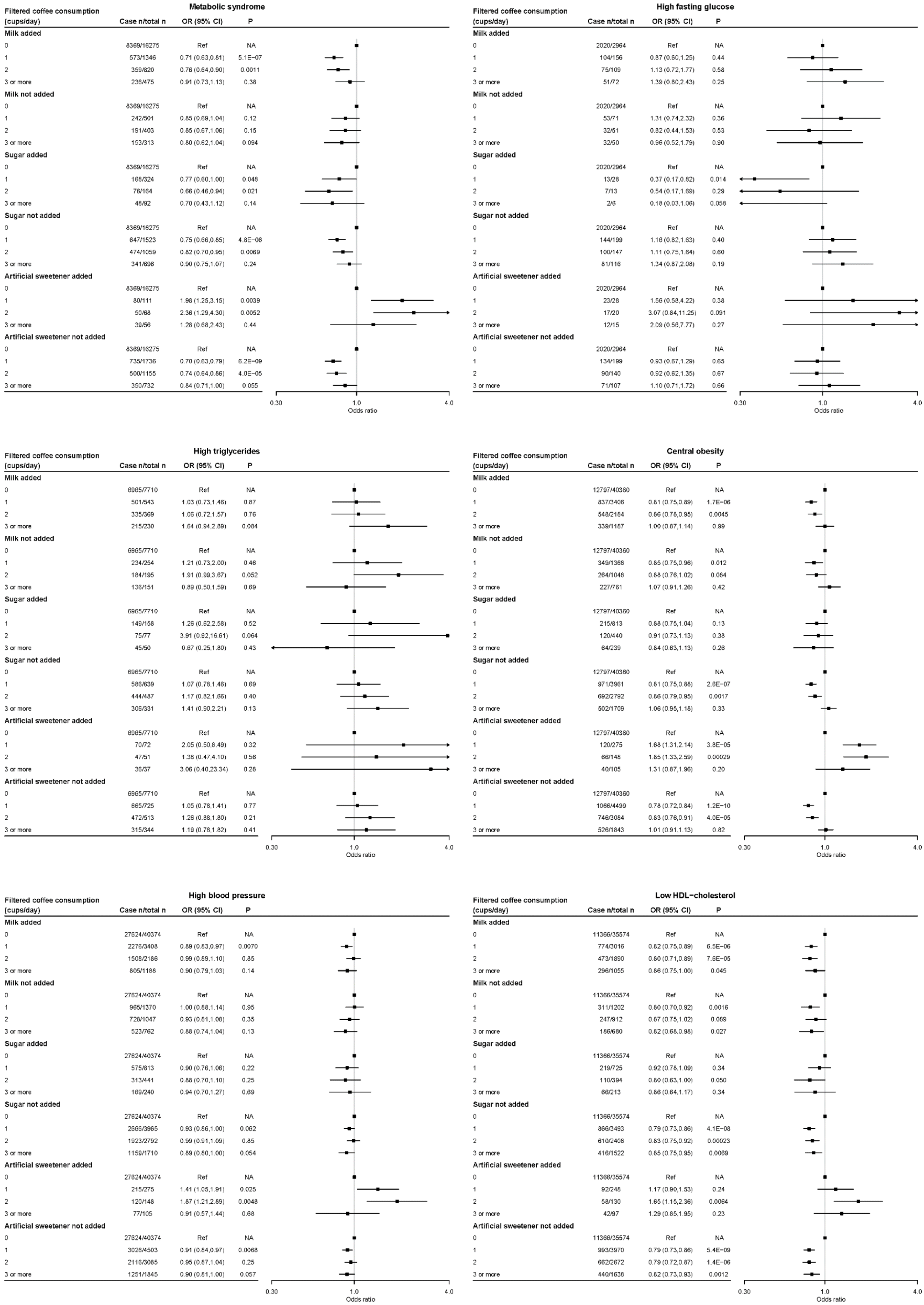

Supplemental figure 6 – Association between filtered coffee consumption and all outcomes, using data were from 24-hour recalls of UK Biobank. All effect estimates were adjusted for age, sex, smoking status, alcohol consumption frequency, vegetable intake, fruit intake, tea intake, physical activity level, and highest qualification obtained. Error bars depict 95% CI.
