# Supplementary material for: Association between coffee consumption and metabolic syndrome: A cross‐sectional and Mendelian randomization study
Source: J Diabetes. 2024 Oct 10;16(10):e70004. doi: 10.1111/1753-0407.70004 (PMC11467012; doi:10.1111/1753-0407.70004)
Supplement: Supplementary file 2 — Data S2. Supporting Information. [file JDB-16-e70004-s001.docx]

**Supplementary Table 1 -** Definition of metabolic syndrome and its component conditions.

| **Conditions** | **Criteria** |
| --- | --- |
| *Metabolic syndrome* | Meeting the criteria for 3 of the following conditions: high fasting glucose, high triglyceride, central obesity, high blood pressure, and Low HDL-cholesterol |
| *High fasting glucose* | Fasting blood glucose level higher than or equal to 100 mg/dL (5.56 mmol/L) or drug treatment of high fasting glucose |
| *Central obesity* | Waist circumference higher than 88 cm in females and 102 cm in males |
| *High triglycerides* | Fasting triglyceride level higher than or equal to 150 mg/dL (1.7 mmol/L) or drug treatment of high triglycerides level |
| *High blood pressure* | Systolic blood pressure higher than or equal to 85 mmHg or diastolic pressure higher than or equal to 130 mmHg or drug treatment of hypertension |
| *Low HDL-cholesterol* | HDL-cholesterol lower than 40 mg/dL (1.0 mmol/L) in males or 50 mg/dL (1.3 mmol/L) in females or drug treatment of low HDL-C levels |

HDL, high density lipoprotein

**Supplemental Table 2 -** List of medications classified as treatment for high fasting glucose.

| Code | Name |
| --- | --- |
| 1140874646 | glipizide |
| 1140874650 | glibenese 5mg tablet |
| 1140874652 | minodiab 2.5mg tablet |
| 1140874658 | gliquidone |
| 1140874660 | glurenorm 30mg tablet |
| 1140874664 | tolazamide |
| 1140874666 | tolanase 100mg tablet |
| 1140874674 | tolbutamide |
| 1140874678 | glyconon 500mg tablet |
| 1140874680 | rastinon 500mg tablet |
| 1140874686 | glucophage 500mg tablet |
| 1140874690 | orabet 500mg tablet |
| 1140874706 | chlorpropamide |
| 1140874712 | diabinese 100mg tablet |
| 1140874716 | glymese 250mg tablet |
| 1140874718 | glibenclamide |
| 1140874724 | daonil 5mg tablet |
| 1140874726 | semi-daonil 2.5mg tablet |
| 1140874728 | euglucon 2.5mg tablet |
| 1140874732 | malix 2.5mg tablet |
| 1140874736 | diabetamide 2.5mg tablet |
| 1140874740 | calabren 2.5mg tablet |
| 1140874744 | gliclazide |
| 1140874746 | diamicron 80mg tablet |
| 1140884600 | Metformin |
| 1141171646 | Pioglitazone |
| 1141171652 | actos 15mg tablet |
| 1141177600 | rosiglitazone |
| 1141177606 | avandia 4mg tablet |
| 1141189090 | rosiglitazone 1mg / metformin 500mg tablet |
| 1141153254 | troglitazone |
| 1140857584 | acetohexamide |
| 1140857494 | glibornuride |
| 1141157284 | glipizide product |
| 1141152590 | glimepiride |
| 1141168660 | repaglinide |
| 1141173882 | nateglinide |
| 1140883066 | insulin product |
| 1140868902 | acarbose |

Codes of medication were extracted from UK Biobank

**Supplemental Table 3 -** List of SNPs used in univariable Mendelian randomization

| **Phenotype** | **SNP** | **Effect allele** | **Reference allele** | **Sample size** | **Beta** | **SE** | **p value** | **Phenotype** | **EAF** | **F-statistic** |
| --- | --- | --- | --- | --- | --- | --- | --- | --- | --- | --- |
| 1 | rs1260326 | T | C | 129417 | -0.04 | 0.01 | 7.14 × 10^-8^ | cups/day | 0.36 | 16.0 |
| 1 | rs1481012 | A | G | 126019 | 0.06 | 0.01 | 8.93 × 10^-8^ | cups/day | 0.89 | 36.0 |
| 1 | rs6968554 | A | G | 124849 | -0.10 | 0.01 | 5.23 × 10^-17^ | cups/day | 0.35 | 100.0 |
| 1 | rs7800944 | T | C | 116417 | -0.05 | 0.01 | 2.29 × 10^-11^ | cups/day | 0.72 | 25.0 |
| 1 | rs17685 | A | G | 115465 | 0.07 | 0.01 | 4.26 × 10^-11^ | cups/day | 0.30 | 49.0 |
| 1 | rs6265 | T | C | 127828 | -0.04 | 0.01 | 2.69 × 10^-6^ | cups/day | 0.18 | 16.0 |
| 1 | rs2472297 | T | C | 116272 | 0.14 | 0.01 | 2.47 × 10^-24^ | cups/day | 0.26 | 196.0 |
| 1 | rs9902453 | A | G | 126819 | -0.03 | 0.01 | 2.44 × 10^-8^ | cups/day | 0.53 | 9.0 |
| 2 | rs6968554 | A | G | 67033 | -0.20 | 0.03 | 7.41 × 10^-15^ | zero/low vs. high | 0.35 | 44.4 |
| 2 | rs17685 | A | G | 63066 | 0.08 | 0.01 | 1.13 × 10^-9^ | zero/low vs. high | 0.27 | 64.0 |
| 2 | rs2470893 | T | C | 62360 | 0.20 | 0.02 | 5.05 × 10^-19^ | zero/low vs. high | 0.32 | 100.0 |

Phenotype 1 refers to coffee consumption in cups per day among coffee consumers only. Phenotype 2 refers to the liability to high coffee consumption (4 cups per day or more), compared with low coffee consumers (1 cup per day or less) or coffee abstainers. SE, standard error. EAF, effect allele frequency.

**Supplemental Table 4** - List of SNPs used in multivariable MR for phenotype 1

| **SNP** | **exposure** | **effect allele** | **other allele** | **beta** | **se** | **p value** |
| --- | --- | --- | --- | --- | --- | --- |
| rs11072518 | cups of coffee per day | T | C | -0.0541 | 0.0079 | 0.000000 |
| rs11634685 | cups of coffee per day | A | C | 0.0466 | 0.0082 | 0.000000 |
| rs11863088 | cups of coffee per day | A | G | 0.2174 | 0.0329 | 0.000000 |
| rs11940694 | cups of coffee per day | A | G | 0.0093 | 0.0079 | 0.241000 |
| rs1229978 | cups of coffee per day | T | C | 0.0011 | 0.0077 | 0.889100 |
| rs12441907 | cups of coffee per day | A | C | -0.0131 | 0.0096 | 0.175400 |
| rs17685 | cups of coffee per day | A | G | 0.0687 | 0.0092 | 0.000000 |
| rs17701498 | cups of coffee per day | T | G | -0.0495 | 0.0077 | 0.000000 |
| rs2040623 | cups of coffee per day | A | C | 0.0875 | 0.0094 | 0.000000 |
| rs2186874 | cups of coffee per day | T | C | -0.0070 | 0.0078 | 0.365200 |
| rs2472297 | cups of coffee per day | T | C | 0.1460 | 0.0101 | 0.000000 |
| rs3001723 | cups of coffee per day | A | G | 0.0025 | 0.0083 | 0.761800 |
| rs351237 | cups of coffee per day | A | G | -0.0567 | 0.0088 | 0.000000 |
| rs4410790 | cups of coffee per day | T | C | -0.1380 | 0.0086 | 0.000000 |
| rs4699680 | cups of coffee per day | A | G | -0.0080 | 0.0184 | 0.664700 |
| rs676388 | cups of coffee per day | T | C | 0.0112 | 0.0081 | 0.168500 |
| rs7187575 | cups of coffee per day | T | C | -0.0108 | 0.0090 | 0.232000 |
| rs7800944 | cups of coffee per day | T | C | -0.0517 | 0.0090 | 0.000000 |
| rs8040372 | cups of coffee per day | A | G | -0.0660 | 0.0082 | 0.000000 |
| rs11072518 | alcoholic drinks per week | T | C | -0.0060 | 0.0031 | 0.052300 |
| rs11634685 | alcoholic drinks per week | A | C | 0.0061 | 0.0031 | 0.048300 |
| rs11863088 | alcoholic drinks per week | A | G | 0.0101 | 0.0081 | 0.209000 |
| rs11940694 | alcoholic drinks per week | A | G | -0.0229 | 0.0030 | 0.000000 |
| rs1229978 | alcoholic drinks per week | T | C | -0.0209 | 0.0030 | 0.000000 |
| rs12441907 | alcoholic drinks per week | A | C | -0.0054 | 0.0038 | 0.153000 |
| rs17685 | alcoholic drinks per week | A | G | -0.0018 | 0.0033 | 0.593000 |
| rs17701498 | alcoholic drinks per week | T | G | -0.0059 | 0.0030 | 0.047400 |
| rs2040623 | alcoholic drinks per week | A | C | 0.0067 | 0.0037 | 0.065700 |
| rs2186874 | alcoholic drinks per week | T | C | -0.0026 | 0.0030 | 0.386000 |
| rs2472297 | alcoholic drinks per week | T | C | 0.0088 | 0.0034 | 0.009370 |
| rs3001723 | alcoholic drinks per week | A | G | 0.0012 | 0.0033 | 0.728000 |
| rs351237 | alcoholic drinks per week | A | G | 0.0000 | 0.0031 | 0.996000 |
| rs4410790 | alcoholic drinks per week | T | C | -0.0045 | 0.0031 | 0.144000 |
| rs4699680 | alcoholic drinks per week | A | G | 0.0421 | 0.0075 | 0.000000 |
| rs676388 | alcoholic drinks per week | T | C | -0.0178 | 0.0030 | 0.000000 |
| rs7187575 | alcoholic drinks per week | T | C | 0.0188 | 0.0032 | 0.000000 |
| rs7800944 | alcoholic drinks per week | T | C | -0.0068 | 0.0033 | 0.038300 |
| rs8040372 | alcoholic drinks per week | A | G | -0.0041 | 0.0030 | 0.174000 |
| rs11072518 | smoke initiation | T | C | 0.0018 | 0.0059 | 0.759000 |
| rs11634685 | smoke initiation | A | C | 0.0043 | 0.0059 | 0.468000 |
| rs11863088 | smoke initiation | A | G | -0.0028 | 0.0160 | 0.861000 |
| rs11940694 | smoke initiation | A | G | 0.0066 | 0.0058 | 0.259000 |
| rs1229978 | smoke initiation | T | C | -0.0010 | 0.0057 | 0.862000 |
| rs12441907 | smoke initiation | A | C | -0.0457 | 0.0072 | 0.000000 |
| rs17685 | smoke initiation | A | G | -0.0052 | 0.0063 | 0.410000 |
| rs17701498 | smoke initiation | T | G | -0.0047 | 0.0057 | 0.411000 |
| rs2040623 | smoke initiation | A | C | 0.0053 | 0.0070 | 0.448000 |
| rs2186874 | smoke initiation | T | C | -0.0420 | 0.0058 | 0.000000 |
| rs2472297 | smoke initiation | T | C | 0.0070 | 0.0065 | 0.279000 |
| rs3001723 | smoke initiation | A | G | 0.0382 | 0.0063 | 0.000000 |
| rs351237 | smoke initiation | A | G | 0.0029 | 0.0058 | 0.622000 |
| rs4410790 | smoke initiation | T | C | 0.0014 | 0.0059 | 0.816000 |
| rs4699680 | smoke initiation | A | G | -0.0135 | 0.0146 | 0.356000 |
| rs676388 | smoke initiation | T | C | -0.0029 | 0.0057 | 0.605000 |
| rs7187575 | smoke initiation | T | C | 0.0141 | 0.0061 | 0.019900 |
| rs7800944 | smoke initiation | T | C | -0.0021 | 0.0063 | 0.739000 |
| rs8040372 | smoke initiation | A | G | 0.0059 | 0.0058 | 0.304000 |

**Supplemental Table 5** - List of SNPs used in multivariable MR for phenotype 2

| **SNP** | **exposure** | **effect allele** | **other allele** | **beta** | **se** | **p value** |
| --- | --- | --- | --- | --- | --- | --- |
| rs11940694 | no/low coffee intake vs. high intake | A | G | -0.0019 | 0.0171 | 0.910100 |
| rs1229978 | no/low coffee intake vs. high intake | T | C | -0.0003 | 0.0163 | 0.984800 |
| rs12441907 | no/low coffee intake vs. high intake | A | C | -0.0507 | 0.0209 | 0.015440 |
| rs17685 | no/low coffee intake vs. high intake | A | G | 0.1095 | 0.0187 | 0.000000 |
| rs2186874 | no/low coffee intake vs. high intake | T | C | -0.0178 | 0.0166 | 0.284300 |
| rs2470893 | no/low coffee intake vs. high intake | T | C | 0.2085 | 0.0181 | 0.000000 |
| rs3001723 | no/low coffee intake vs. high intake | A | G | 0.0098 | 0.0178 | 0.581200 |
| rs4699680 | no/low coffee intake vs. high intake | A | G | -0.0227 | 0.0397 | 0.567600 |
| rs6265 | no/low coffee intake vs. high intake | T | C | -0.1154 | 0.0211 | 0.000000 |
| rs676388 | no/low coffee intake vs. high intake | T | C | 0.0405 | 0.0168 | 0.015790 |
| rs6968554 | no/low coffee intake vs. high intake | A | G | -0.2303 | 0.0178 | 0.000000 |
| rs7791070 | no/low coffee intake vs. high intake | T | C | 0.1697 | 0.0207 | 0.000000 |
| rs8040372 | no/low coffee intake vs. high intake | A | G | -0.1251 | 0.0173 | 0.000000 |
| rs11940694 | smoke initiation | A | G | 0.0066 | 0.0058 | 0.259000 |
| rs1229978 | smoke initiation | T | C | -0.0010 | 0.0057 | 0.862000 |
| rs12441907 | smoke initiation | A | C | -0.0457 | 0.0072 | 0.000000 |
| rs17685 | smoke initiation | A | G | -0.0052 | 0.0063 | 0.410000 |
| rs2186874 | smoke initiation | T | C | -0.0420 | 0.0058 | 0.000000 |
| rs2470893 | smoke initiation | T | C | 0.0074 | 0.0061 | 0.225000 |
| rs3001723 | smoke initiation | A | G | 0.0382 | 0.0063 | 0.000000 |
| rs4699680 | smoke initiation | A | G | -0.0135 | 0.0146 | 0.356000 |
| rs6265 | smoke initiation | T | C | -0.0245 | 0.0074 | 0.000920 |
| rs676388 | smoke initiation | T | C | -0.0029 | 0.0057 | 0.605000 |
| rs6968554 | smoke initiation | A | G | 0.0016 | 0.0059 | 0.787000 |
| rs7791070 | smoke initiation | T | C | -0.0023 | 0.0069 | 0.741000 |
| rs8040372 | smoke initiation | A | G | 0.0059 | 0.0058 | 0.304000 |
| rs11940694 | alcoholic drinks per week | A | G | -0.0229 | 0.0030 | 0.000000 |
| rs1229978 | alcoholic drinks per week | T | C | -0.0209 | 0.0030 | 0.000000 |
| rs12441907 | alcoholic drinks per week | A | C | -0.0054 | 0.0038 | 0.153000 |
| rs17685 | alcoholic drinks per week | A | G | -0.0018 | 0.0033 | 0.593000 |
| rs2186874 | alcoholic drinks per week | T | C | -0.0026 | 0.0030 | 0.386000 |
| rs2470893 | alcoholic drinks per week | T | C | 0.0076 | 0.0032 | 0.017600 |
| rs3001723 | alcoholic drinks per week | A | G | 0.0012 | 0.0033 | 0.728000 |
| rs4699680 | alcoholic drinks per week | A | G | 0.0421 | 0.0075 | 0.000000 |
| rs6265 | alcoholic drinks per week | T | C | -0.0106 | 0.0039 | 0.006120 |
| rs676388 | alcoholic drinks per week | T | C | -0.0178 | 0.0030 | 0.000000 |
| rs6968554 | alcoholic drinks per week | A | G | -0.0044 | 0.0031 | 0.155000 |
| rs7791070 | alcoholic drinks per week | T | C | 0.0070 | 0.0036 | 0.053200 |
| rs8040372 | alcoholic drinks per week | A | G | -0.0041 | 0.0030 | 0.174000 |

**Supplemental Table 6** - Full results of Mendelian randomization analysis regarding coffee consumption among coffee consumers only (phenotype 1).

|  |  |  | **Inverse-variance weighted** | | | | **MR-Egger** | | | | | **Weighted median** | | | |
| --- | --- | --- | --- | --- | --- | --- | --- | --- | --- | --- | --- | --- | --- | --- | --- |
| **Outcome** | ***n*** | ***n*_snp_** | **OR** | **95% CI** | ***p*-value** | **Cochran's *Q* *p*-value** | **OR** | **95% CI** | ***p*-value** | **Intercept term** | ***p*-value of intercept term** | **OR** | **95% CI** | ***p*-value** |  |
| *Metabolic syndrome* | 164235 | 8 | 1.141 | (0.723, 1.801) | 0.57112 | <0.00001 | 2.230 | (0.989, 5.028) | 0.101253 | -0.05630 | 0.11424 | 1.218 | (0.968, 1.533) | 0.09302 |  |
| *High fasting glucose* | 35819 | 8 | 1.787 | (0.937, 3.409) | 0.07803 | 0.00002 | 1.080 | (0.275, 4.237) | 0.915319 | 0.04229 | 0.44094 | 1.513 | (1.05, 2.181) | 0.02648 |  |
| *High triglycerides* | 82129 | 8 | 0.942 | (0.296, 2.997) | 0.91905 | <0.00001 | 5.087 | (0.635, 40.759) | 0.176418 | -0.14185 | 0.11963 | 1.734 | (1.146, 2.624) | 0.00924 |  |
| *Central obesity* | 401572 | 8 | 1.345 | (1.100, 1.645) | 0.00391 | <0.00001 | 1.336 | (0.852, 2.093) | 0.253258 | 0.00058 | 0.97372 | 1.314 | (1.179, 1.466) | <0.00001 |  |
| *High blood pressure* | 402040 | 8 | 0.974 | (0.815, 1.163) | 0.77022 | 0.00170 | 1.227 | (0.878, 1.716) | 0.276380 | -0.01945 | 0.17328 | 0.966 | (0.829, 1.125) | 0.65500 |  |
| *Low HDL-cholesterol* | 359771 | 8 | 0.983 | (0.675, 1.433) | 0.93038 | <0.00001 | 1.573 | (0.764, 3.24) | 0.264946 | -0.03949 | 0.19533 | 1.049 | (0.887, 1.24) | 0.57529 |  |

**Supplemental Table 7 -** Full results of Mendelian randomization analysis regarding zero/low vs. high coffee consumption (phenotype 2).

|  |  |  | **Inverse-variance weighted** | | | | **MR-Egger** | | | | | **Weighted median** | | | |
| --- | --- | --- | --- | --- | --- | --- | --- | --- | --- | --- | --- | --- | --- | --- | --- |
| **Outcome** | ***n*** | ***n*_snp_** | **OR** | **95% CI** | ***p*-value** | **Cochran's *Q* *p*-value** | **OR** | **95% CI** | ***p*-value** | **Intercept term** | **p-value of intercept term** | **OR** | **95% CI** | ***p*-value** |  |
| *Metabolic syndrome* | 164235 | 3 | 1.203 | (0.97, 1.493) | 0.09208 | 0.00444 | 1.136 | (0.415, 3.109) | 0.84458 | 0.01058 | 0.92594 | 1.219 | (1.047, 1.419) | 0.01059 |  |
| *High fasting glucose* | 35819 | 3 | 1.237 | (0.999, 1.531) | 0.05094 | 0.26421 | 0.807 | (0.435, 1.495) | 0.61854 | 0.07903 | 0.38989 | 1.205 | (0.980, 1.481) | 0.07712 |  |
| *High triglycerides* | 82129 | 3 | 1.274 | (0.856, 1.897) | 0.23226 | 0.00687 | 0.896 | (0.156, 5.149) | 0.92173 | 0.06535 | 0.75012 | 1.238 | (0.960, 1.597) | 0.09926 |  |
| *Central obesity* | 401572 | 3 | 1.175 | (1.112, 1.242) | <0.00001 | 0.49689 | 1.058 | (0.881, 1.271) | 0.65536 | 0.01948 | 0.44708 | 1.166 | (1.086, 1.252) | 0.00002 |  |
| *High blood pressure* | 402040 | 3 | 1.002 | (0.876, 1.147) | 0.97255 | 0.00793 | 1.010 | (0.534, 1.908) | 0.98096 | -0.00136 | 0.98491 | 0.997 | (0.903, 1.102) | 0.95798 |  |
| *Low HDL-cholesterol* | 359771 | 3 | 1.085 | (0.857, 1.373) | 0.49874 | <0.00001 | 1.025 | (0.342, 3.072) | 0.97218 | 0.01050 | 0.93251 | 1.085 | (0.964, 1.22) | 0.17589 |  |

**Supplemental Table 8** - Full results of multivariable Mendelian randomization analysis regarding coffee consumption among coffee consumers only (phenotype 1) after adjusting for alcohol consumption and liability to smoke initiation

|  |  | **Inverse-variance weighted** | | | | **MR-Egger** | | | |
| --- | --- | --- | --- | --- | --- | --- | --- | --- | --- |
| **Outcome** | ***n*_snp_** | **OR** | **95% CI** | ***p*-value** | **Cochran's *Q* *p*-value** | **OR** | **95% CI** | ***p*-value** | ***p*-value of intercept term** |
| *Metabolic syndrome* | 19 | 1.113 | (0.848, 1.461) | 0.44028 | <0.00001 | 1.857 | (1.060, 3.251) | 0.03045 | 0.04563 |
| *High fasting glucose* | 19 | 1.380 | (1.104, 1.724) | 0.00466 | 0.56097 | 1.235 | (0.749, 2.038) | 0.40837 | 0.62916 |
| *High triglycerides* | 19 | 1.191 | (0.782, 1.814) | 0.41541 | <0.00001 | 2.780 | (1.189, 6.500) | 0.01834 | 0.02895 |
| *Central obesity* | 19 | 1.281 | (1.106, 1.483) | 0.00094 | <0.00001 | 1.076 | (0.777, 1.49) | 0.65989 | 0.24084 |
| *High blood pressure* | 19 | 0.896 | (0.721, 1.112) | 0.31883 | <0.00001 | 1.390 | (0.897, 2.152) | 0.14032 | 0.02786 |
| *Low HDL-cholesterol* | 19 | 1.007 | (0.818, 1.240) | 0.94902 | <0.00001 | 1.486 | (0.967, 2.284) | 0.07107 | 0.04760 |

**Supplemental Table 9** - Full results of multivariable Mendelian randomization analysis regarding zero/low vs high coffee consumption (phenotype 2) after adjusting for alcohol consumption and liability to smoke initiation

|  |  | **Inverse-variance weighted** | | | | **MR-Egger** | | | |
| --- | --- | --- | --- | --- | --- | --- | --- | --- | --- |
| **Outcome** | ***n*_snp_** | **OR** | **95% CI** | ***p*-value** | **Cochran's *Q* *p*-value** | **OR** | **95% CI** | ***p*-value** | ***p*-value of intercept term** |
| *Metabolic syndrome* | 13 | 1.126 | (0.999, 1.270) | 0.05202 | 0.00205 | 1.180 | (0.984, 1.415) | 0.07380 | 0.49206 |
| *High fasting glucose* | 13 | 1.215 | (1.052, 1.403) | 0.00812 | 0.71573 | 1.225 | (0.991, 1.514) | 0.06071 | 0.91555 |
| *High triglycerides* | 13 | 1.166 | (0.955, 1.424) | 0.13235 | 0.02394 | 1.362 | (1.031, 1.798) | 0.02952 | 0.13613 |
| *Central obesity* | 13 | 1.155 | (1.086, 1.227) | <0.00001 | 0.02852 | 1.078 | (1.002, 1.159) | 0.04276 | 0.01133 |
| *High blood pressure* | 13 | 0.978 | (0.885, 1.081) | 0.66740 | <0.00001 | 1.047 | (0.908, 1.208) | 0.52863 | 0.20513 |
| *Low HDL-cholesterol* | 13 | 1.046 | (0.935, 1.170) | 0.43304 | <0.00001 | 1.083 | (0.912, 1.285) | 0.36483 | 0.59231 |
